# Machine learning–based triage to identify low-severity patients with a short discharge length of stay in emergency department

**DOI:** 10.1186/s12873-022-00632-6

**Published:** 2022-05-20

**Authors:** Yu-Hsin Chang, Hong-Mo Shih, Jia-En Wu, Fen-Wei Huang, Wei-Kung Chen, Dar-Min Chen, Yu-Ting Chung, Charles C. N. Wang

**Affiliations:** 1 Department of Emergency Medicine, China Medical University Hospital, No. 2, Yude Rd., North Dist., Taichung City, 40447 Taiwan; 2 Department of Bioinformatics and Medical Engineering, Asia University, No. 500, Liufeng Rd., Wufeng Dist., Taichung City, 413305 Taiwan; 3 Department of Medical Research, China Medical University Hospital, No. 2, Yude Rd., North Dist., Taichung City, 40447 Taiwan; 4 Department of Emergency Medicine, Asia University Hospital, No. 222, Fuxin Rd., Wufeng Dist., Taichung City, 413505 Taiwan

**Keywords:** Emergency department, Machine learning, Triage, Discharge length of stay, Decision-making support, Streaming

## Abstract

**Background:**

Overcrowding in emergency departments (ED) is a critical problem worldwide, and streaming can alleviate crowding to improve patient flows. Among triage scales, patients labeled as “triage level 3” or “urgent” generally comprise the majority, but there is no uniform criterion for classifying low-severity patients in this diverse population. Our aim is to establish a machine learning model for prediction of low-severity patients with short discharge length of stay (DLOS) in ED.

**Methods:**

This was a retrospective study in the ED of China Medical University Hospital (CMUH) and Asia University Hospital (AUH) in Taiwan. Adult patients (aged over 20 years) with Taiwan Triage Acuity Scale level 3 were enrolled between 2018 and 2019. We used available information during triage to establish a machine learning model that can predict low-severity patients with short DLOS. To achieve this goal, we trained five models—CatBoost, XGBoost, decision tree, random forest, and logistic regression—by using large ED visit data and examined their performance in internal and external validation.

**Results:**

For internal validation in CMUH, 33,986 patients (75.9%) had a short DLOS (shorter than 4 h), and for external validation in AUH, there were 13,269 (82.7%) patients with short DLOS. The best prediction model was CatBoost in internal validation, and area under the receiver operating cha racteristic curve (AUC) was 0.755 (95% confidence interval (CI): 0.743–0.767). Under the same threshold, XGBoost yielded the best performance, with an AUC value of 0.761 (95% CI: 0.742- 0.765) in external validation.

**Conclusions:**

This is the first study to establish a machine learning model by applying triage information alone for prediction of short DLOS in ED with both internal and external validation. In future work, the models could be developed as an assisting tool in real-time triage to identify low-severity patients as fast track candidates.

## Background

Surging emergency visits are a critical problem and cause overcrowding in emergency departments (EDs) worldwide. To improve patient flows, streaming is a possible resolution for alleviating crowding [1, 2]. The first step of streaming occurs during triage, when medical staffs stratify patients according to their urgency of need for medical care. Formal five-level triage scales include the Canadian Triage and Acuity Scale (CTAS), the Emergency Severity Index (ESI), and the Manchester Triage System and Australasian Triage System [3–6]. In Taiwan, the five-level Taiwan Triage and Acuity Scale (TTAS) was adapted from the CTAS and was developed as a computerized system that has been used in all emergency rooms [7].

Among triage scales, patients labeled as triage level 3 or “urgent” generally comprise the major population [8–13]. Studies have reported a large diversity in disposition and resource consumption in this population. Some patients may experience early mortality, be admitted to the intensive care unit (ICU) or general ward, or be discharged after various length of stay (LOS) [11, 14, 15]. Arya et al. emphasized the importance of splitting high-variability patients in ESI level 3 to improve ED crowding [16]. To further differentiate patients with lower severity from this major population, various criteria have been used in research, and the criteria generally include stable vital signs, ambulatory status, and specific chief complaints [13, 16–18]. These lower severity patients were classified the same as those of level 4 and level 5 and regarded as candidates for fast tract. In addition, Casalino et al. reported that patients with shorter LOS (greater than 160 min) was associated with less medical and nurse resource consumption [19]. Therefore, predicting a short discharge LOS (DLOS) may be a method of categorizing low-severity patients for further streaming.

Studies have mainly focused on identifying attributes to predict prolonged ED LOS using statistical techniques [12, 19–21]. Furthermore, d’Etienne et al., Gill et al., and Rahman et al. have introduced machine learning models to predict prolonged LOS [22–24]. However, in these studies, features for machine learning or statistical techniques have been derived from triage information and from physician orders, which signifies that their prediction models can be used only after physician assessment. This is unsuitable for triage where assessment was executed only by nurses.

In this study, we used available information during triage to establish a machine learning model that can predict low-severity patients with short DLOS in a population with TTAS level 3. To achieve this goal, we trained 5 machine learning models by using large ED visit data and examined the performance in internal and external validation. Our prediction model can be used in real-time triage for streaming to identify low-severity patients as fast track candidates.

## Methods

### Study design, setting and participants

This study was approved by the Institutional Review Board of China Medical University (CMUH109-REC1-021). All methods were performed in accordance with relevant guidelines, and individual informed consent was waived because of the study design. It was a retrospective research by applying ED datasets of two hospitals in Taiwan. Dataset since Jan. 2018 to Dec. 2018 from China Medical University Hospital (CMUH) was used for model construction and internal validation, and dataset since Jan. 2018 to Dec. 2019 from Asia University Hospital (AUH) was applied for external validation. CMUH is a 1700-bed, urban, academic, tertiary care hospital with approximately 150 000 to 160 000 ED visits annually. AUH is a 482-bed regional hospital, and annual ED visits are around 36,000 persons.

The computerized TTAS system evaluates (a) trauma or nontrauma; (b) chief complaints; (C) injury mechanisms; and (d) first-order modifiers, such as vital signs (including degree of respiratory distress, hemodynamic stability, conscious level, body temperature, and pain severity), to determine the triage level. Secondary order modifiers are used if the triage level cannot be determined according to these variables. The 2 main systems of TTAS are the traumatic and nontraumatic systems. The nontraumatic system contains 13 categories with 125 chief complaints (pulmonary, cardiovascular, digestive, neurological, musculoskeletal, genitourinary, ear, nose, and throat–related, ophthalmologic, dermatologic, obstetric and gynecologic, psychiatric, general, and other disorders) [25].

Adult patients (aged over 20 years) with TTAS level 3 were enrolled, and we excluded patients with the following criteria: 1) death on arrival, 2) trauma, 3) having left without being seen, 4) discharge against medical advice, 5) admission to either ward or ICU, 6) transfer to another hospital, 7) missing information, and 8) inconsistent data (i.e., systolic blood pressure (SBP) > 300 mmHg or < 30 mmHg, diastolic blood pressure (DBP) > 300 mmHg, SBP < DBP, pulse rate > 300/min or < 20/min, respiratory rate > 60/min, body temperature > 45 °C or < 30 °C, and body mass index (BMI) > 150 or < 5).

### Data collection

The triage data were recorded routinely by each triage nurse and were extracted from electronic databases in two hospitals. The information included age, gender, BMI, vital signs, consciousness, indwelling tube, whether the patient was transferred and the facility the patient was transferred from, mode of arrival, bed request, comorbidity, pregnancy, frequency of intensive ED visits (> 2 times a week or > 3 times a month), 72-h unscheduled returns, and the system of chief complaint.

### Machine learning models

In this study, we used the following 5 machine learning classification models: CatBoost, XGBoost, decision tree (DT), random forest (RF), and logistic regression (LR) [26–30]. We explored the parameter space and common variations for each machine learning classification model as thoroughly as computationally feasible. XGBoost uses no weighted sampling techniques, which slows its splitting process compared with that of gradient-based one-side sampling and minimal variance sampling (MVS). CatBoost offers a new technique called MVS, which is a weighted sampling version of stochastic gradient boosting. CatBoost-weighted sampling happens at the tree-level and not at the split-level. The observations for each boosting tree are sampled to maximize the accuracy of split scoring. DT is one of the earliest and most prominent machine learning based on decision logics. DTs have multiple levels in which the first or topmost node is called the root node. All internal nodes represent tests on input variables or attributes. Depending on the test outcome, the classification algorithm branches toward the appropriate child node where the process of testing and branching repeats until it reaches the leaf node. RF is a classification algorithm that works by forming multiple DTs to train and test the classes it outputs. A DT effectively learns the characteristics of simple decision rules that are extracted from the data. The deeper the tree, the more complex the rules and the healthier the decision. RFs overcome problematic trees that are over-adapted to decision-making. LR can be considered as an extension of ordinary regression and can model only a dichotomous variable that typically represents the occurrence or nonoccurrence of an event. LR helps in finding the probability that a new instance belongs to a certain class. Supervised learning is mainly used to learn a model by learning the training data of multiple features and predicting the result of the target variable through the model. The model is represented by a mathematical function; furthermore, by using the objective function of predicting Y from a given X—wherein the parameters of the model are learned, adjusted from the data, and depend on the predicted value—we can classify problem types into regression or classification.6

The dataset of CMUH was divided into 2 subsets, where 80% of the sample was for the training set and the remaining 20% sample was used to test the trained mode. Besides, all the data from AUH was used for the externa validation. To indicate prediction performance, we computed the receiver operating characteristics (ROC) curve, the area under the ROC curve (AUC), sensitivity, specificity, positive predicted value, and negative predictive value. All ML algorithms and competed performance analysis were conducted using scikit-learn and the XGBoost library.

### Feature selection

We filtered the data to the remaining 32 pieces of inspection information and began to train and evaluate our model, which we then discussed with another doctor who picked out the least used feature. In general, a few or several variables are commonly used in machine learning predictive models and are not associated with the response. In practice, including such irrelevant variables leads to unnecessary complexity in the resulting model. Therefore, in this study, we used the popular feature importance selection tool scikit-learn to choose the most effective attributes in classifying training data. This algorithm assesses the weight of each variable by evaluating the Gini index regarding the outcome and then ranks the variables according to their weights.

### Parameter optimization

Machine learning algorithms involve a few hyperparameters that must be fixed before the algorithms are run. Grid search is often used in the machine learning literature, and it is used to optimize the hyperparameters of the machine learning model. Grid search is a traversal of each intersection in the grid to find the best combination. The dimension of the grid is the number of super parameters. If there are k parameters and each parameter has candidates, we must traverse k × m combinations. Grid search yields good results at the expense of very slow implementation efficiency. Bergstra and Bengio noted that random search is more efficient than grid search [31]. In this study, only when the number of searches was the same were the random search results the same as the web search results. Therefore, the web search speed was slow, but the optimization result was better. For the detailed hyper-parameterization of the algorithms, please refer to the scikit-learn documentation [32].

### Outcomes

The outcome was a short DLOS of < 4 h in the ED, and the DLOS was measured as the time interval between being registered in the ED triage and being discharged from the ED.

## Results

In 2018, CMUH had a total of 127 749 nontraumatic ED visits, including those from 92 528 adult patients. Of these, 58 743 adults without traumas with TTAS level 3 were enrolled. We excluded 10 199 visits with admission to either ward or ICU, 9 visits in which the patient died in the ED, 69 visits in which patients left without being seen, 296 visits in which patients escaped, 2849 visits in which patients left against medical advice, 383 visits in which patients were transferred to other hospitals or outpatient department, and 99 visits in which patients had an indeterminable disposition. In total, 44 839 patients were discharged directly from the ED. The remaining patients had 64 records (0.14%) with either inconsistent or missing data. The final analytic cohort comprised 44 775 ED visits. We used 28 656 patients (64%) in the training set, 7164 patients (16%) in the validation set, and 8955 patients (20%) in the test set. During 2018 to 2019 in AUH, there were 44,947 adult patients without trauma, and 29,730 patients belonged to TTAS level 3. We then further excluded 5865 patients admitting to either ward or ICU, 1 patient expired in ED, 2 patients left without being seen, 29 escaped patients, 571 patients discharged against medical advice, 522 patients transferred to other hospitals or outpatient department, and 157 patients with ambiguous disposition. Among the remaining 22,587 patients directly discharged from ED, there were 6540 patients (28.95%) with either missing or inconsistent data. The final cohort was composed of 16,047 patients. Table 1 shows the demographic characteristics of patients from CMUH and AUH datasets. In CMUH, 33 986 patients (75.9%) had short DLOS (< 4 h), and the number of female patients (58.4%) was greater than that of male patients. The mean age of the entire cohort was 45.7 years, and most patients visited the ED directly without transfer (95.1%) or using an ambulance (94.7%). Gastrointestinal, neurological, and general complaints accounted for the majority of complaints (62.95%). In AUH, 13,269 patients (82.7%) had short DLOS, and the top 5 most common system of complaints were the same with those in CMUH.

**Table 1:** Demographic characteristics of ED visits in CMUH and AUH

Variables	CMUH	AUH
	*N* = 44,775	*N* = 16,047
Age, mean ± SD	45.65 ± 17.86	46.39 ± 18.21
Sex-female, No. (%)	26,185 (58.48)	8807 (54.88)
Body mass index, mean ± SD	23.76 ± 4.56	24.36 ± 4.49
Respiratory rate, mean ± SD	19.88 ± 1.24	18.45 ± 2.27
SBP (mmHg), mean ± SD	136.93 ± 23.50	138.33 ± 24.56
DBP (mmHg), mean ± SD	86.70 ± 16.05	80.17 ± 15.35
Heart rate (bpm), mean ± SD	89.36 ± 17.43	89.05 ± 18.39
Body temperature (℃), mean ± SD	36.95 ± 0.80	37.08 ± 0.87
Consciousness, No. (%)		
Alert (6)	44,616 (99.64)	15,987 (99.63)
Voice (5)	100 (0.22)	41 (0.26)
Pain (4)	41 (0.09)	12 (0.07)
Unresponsive (1–3)	18 (0.04)	7 (0.04)
Tracheostomy, No. (%)	8 (0.02)	1 (0.01)
Drainage tube, No. (%)	16 (0.04)	1 (0.01)
Nasogastric tube, No. (%)	157 (0.35)	15 (0.09)
FOLEY catheter, No. (%)	209 (0.47)	24 (0.15)
Transferred, No. (%)	2212 (4.94)	515 (3.21)
Hospitals	418 (0.93)	344 (2.14)
Clinics	1685 (3.76)	153 (0.93)
Nursing home	34 (0.08)	18 (0.11)
Others	75 (0.17)	0 (0.00)
Arrival by ambulance, No. (%)	2364 (5.28)	377 (2.34)
Bed request, No (%)	3412 (7.62)	999 (6.22)
Comorbidity, No. (%)		
Diabetes mellitus	4963 (11.08)	1963 (12.23)
Hypertension	8747 (19.54)	3544 (24.58)
Unknown heart disease	3545 (7.92)	893 (5.56)
Congestive heart failure	144 (0.32)	49 (3.05)
Ischemic heart disease	417 (0.93)	50 (0.31)
End-stage renal disease	672 (1.50)	262 (1.63)
Liver cirrhosis	413 (0.92)	143 (0.89)
COPD	155 (0.35)	85 (0.52)
Cancer	2969 (6.63)	600 (3.74)
Pregnancy, No. (%)	801 (1.79)	173 (1.08)
ED visits over twice in a week, No. (%)	2930 (6.54)	1094 (6.82)
ED visits over 3 times in a month, No. (%)	1634 (3.65)	661 (4.12)
72-h ED return, No. (%)	1420 (3.17)	504 (3.14)
Common system of complaints, No. (%)		
Gastrointestinal-related	15,097 (33.72)	5199 (32.40)
Neurological-related	7838 (17.51)	3186 (19.85)
General	5247 (11.72)	2162 (13.47)
Cardiovascular-related	4278 (9.55)	1460 (9.10)
Urological-related	2978 (6.65)	1143 (7.12)
**DLOS**, No. (%)		
≧ 4 h	10,789 (24.1%)	2778 (17.31)
< 4 h	33,986 (75.9%)	13,269 (82.69)

*Abbreviation*: *SD* Standard deviation, *SBP* Systolic blood pressure, *DBP* Diastolic blood pressure, *COPD* Chronic obstructive pulmonary disease, *ED* Emergency department, *DLOS* Discharge length of stay, *CMUH* China Medical University Hospital, *AUH* Asia University Hospital

### Prediction of a short DLOS

#### (a) Internal validation

**Fig. 1:**
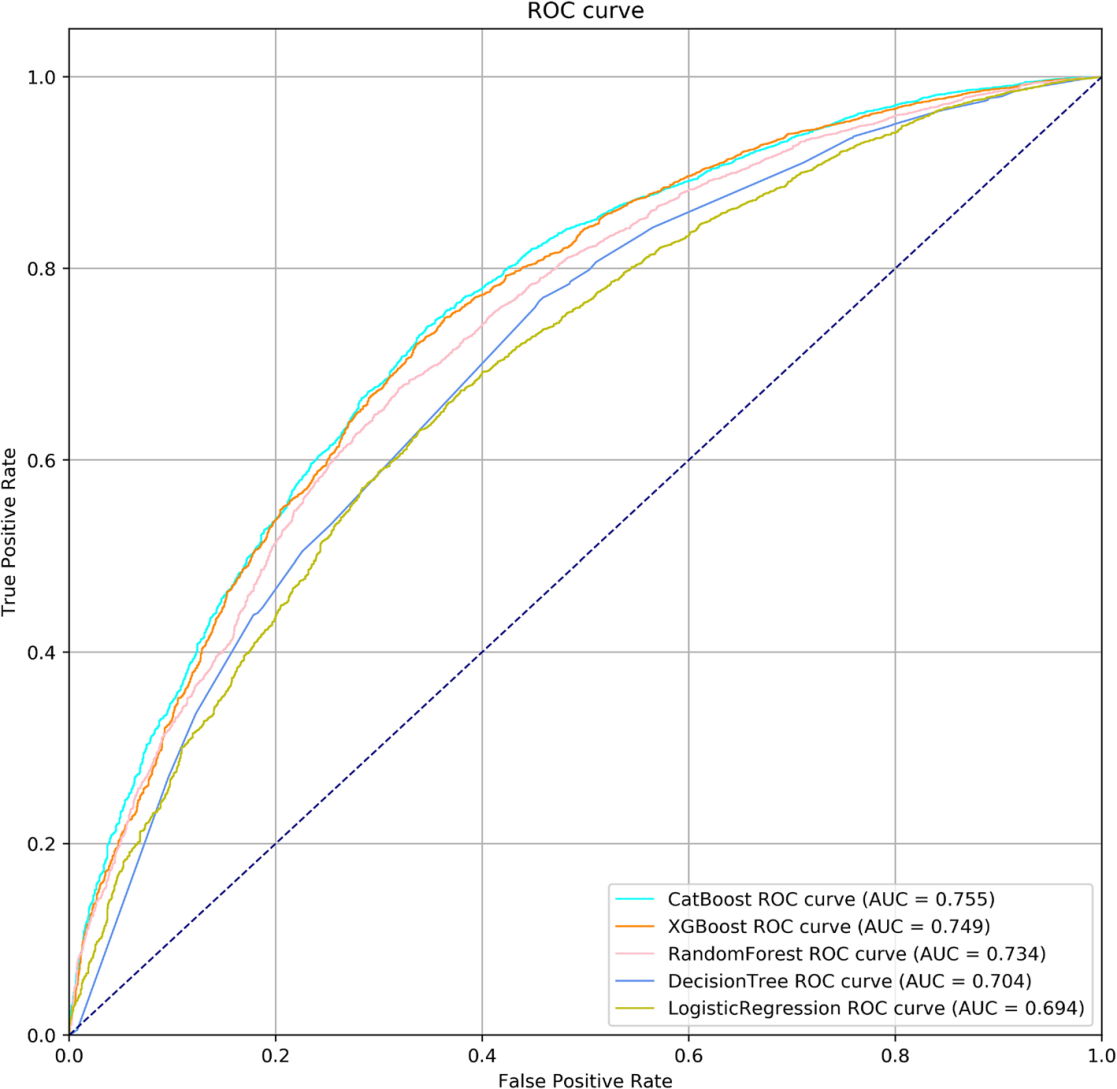
Receiver operating characteristic (ROC) curves. ROC curves of machine learning models for short discharge lengths of stay (DLOS) in the test set of internal validation

The performance of the 5 models in the test set are illustrated in Fig. 1 and Table 2. The AUC for 5 models (CatBoost, XGBoost, DT, RF, and LR) were 0.755 (95% confidence interval (CI): 0.743–0.767), 0.749 (95% CI: 0.736–0.761), 0.704 (95% CI: 0.691–0.717), 0.733 (95% CI: 0.720–0.745), and 0.694 (95% CI: 0.681–0.707), respectively. CatBoost yielded the best AUC, the specificity was 83.12% (95% CI: 81.43–84.72%), and the positive predictive value (PPV) was 90.64% (95% CI: 89.77–91.45%).

**Table 2:** Prediction performance of internal validation in CMUH

Model	AUC	Sensitivity	Specificity	PPV	NPV
**CatBoost**	**0.755 (0.743–0.767)**	**48.70% (47.52–49.89)**	**83.12% (81.43–84.72)**	**90.64% (89.77–91.45)**	**32.56% (31.91–33.23)**
XGBoost	0.749 (0.736–0.761)	51.50% (50.31–52.69)	81.08% (79.32–82.75)	90.13% (89.28–90.92)	33.25% (32.55–33.97)
Random Forest	0.733 (0.720–0.745)	33.80% (34.67–36.95)	88.18% (86.71–89.55)	91.04% (90.00–91.99)	29.05% (28.56–29.54)
Decision tree	0.704 (0.691–0.717)	44.05% (42.87–45.23)	81.76% (80.02–83.41)	89.02% (88.05–89.91)	30.34% (29.72–30.96)
Logistic regression	0.694 (0.681–0.707)	28.71% (27.65–29.80)	89.80% (88.46–91.03)	89.80% (88.55–90.93)	27.13% (28.30–29.14)

*Abbreviation*: *AUC* Area under the receiver operating characteristic curve, *PPV* Positive predictive value, *NPV* Negative predictive value, *CMUH* China Medical University Hospital

**Fig. 2:**
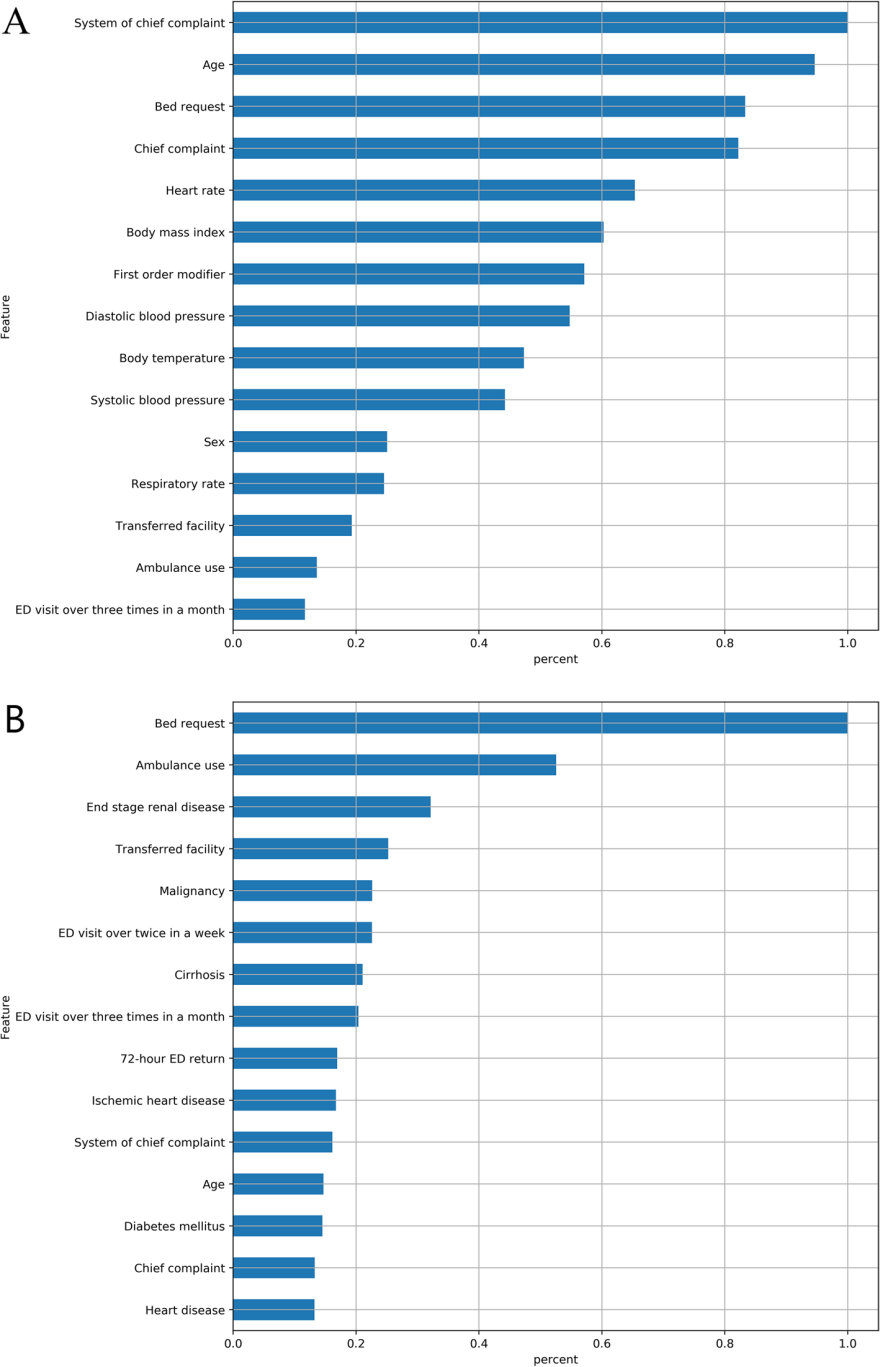
Relative importance of top 15 predictive features. Measurement was scaled with a maximum value of 1.0 in **A**) the CATboost model and **B**) the Xgboost model

From the CatBoost model, the 5 most crucial predictors were ambulation, chief complaint, system of chief complaint, first-order modifier, and whether the patient was transferred (Fig. 2A). The variable importance ranking up to the fifth was different in the XGBoost model. BMI was the most important variable, followed by heart rate, DBP, SBP, and chief complaint (Fig. 2B).

Table 3 presents the performance under a different threshold (0.80–0.95) in each model, and an assumption of per 100 patients for screening the model were used to simulate the real situation in the ED. Under a threshold of 0.85, CatBoost had 83.12% specificity and 90.64% PPV, and 41.40 patients were identified to have a short DLOS. Of these, 37.52 was true and 3.88 was false. The specificity and PPV increased to 99.90% and 98.68%, respectively, and only 1.70 patients were identified with a short DLOS under CatBoost when we adopted a threshold of 0.95. Different thresholds decided the performance of each model.

**Table 3:** Operational performance among various thresholds in internal validation

Threshold and models	Sensitivity	Specificity	PPV	Identification per 100 ED visits		
				Total	True	False
**0.80**						
CatBoost	65.52%	71.98%	88.70%	56.90	50.47	6.43
XGBoost	66.26%	71.01%	88.47%	57.70	51.04	6.66
Decision tree	53.23%	74.71%	87.60%	46.80	40.99	5.81
Random forest	53.86%	78.26%	89.26%	46.50	41.51	4.99
Logistic regression	58.47%	70.09%	86.77%	51.90	45.03	6.87
**0.85**						
CatBoost	48.70%	83.12%	90.64%	41.40	37.52	3.88
XGBoost	51.50%	81.08%	90.13%	44.00	39.66	4.34
Decision tree	44.05%	81.76%	89.02%	38.10	33.92	4.18
Random forest	35.18%	88.42%	91.07%	29.80	27.14	2.66
Logistic regression	28.19%	89.40%	89.92%	24.20	21.76	2.44
**0.90**						
CatBoost	24.34%	94.60%	93.80%	20.00	18.76	1.24
XGBoost	30.44%	91.00%	91.90%	25.50	23.44	2.06
Decision tree	26.86%	90.42%	90.39%	22.90	20.70	2.20
Random forest	0.00%	N/A	N/A	0.00	0.00	0.00
Logistic regression	1.61%	99.90%	98.23%	1.30	1.28	0.02
**0.95**						
CatBoost	2.17%	99.90%	98.68%	1.70	1.68	0.02
XGBoost	7.29%	98.98%	95.99%	5.90	5.66	0.24
Decision tree	0.04%	99.81%	42.86%	0.90	0.39	0.51
Random forest	0.00%	N/A	N/A	0.00	0.00	0.00
Logistic regression	0.00%	N/A	N/A	0.00	0.00	0.00

*Abbreviation*: *PPV* Positive predictive value

### (b) External validation

Under the same threshold of 0.85, prediction performance of dataset in AUH was reported in Table 4. XGBoost yielded the best performance and had an AUC value of 0.761 (CI: 0.742–0.765). The sensitivity was 57.64% (CI: 57.16–58.12) and specificity was 81.43% (CI: 80.62–82.22).

**Table 4:** Prediction performance of external validation in AUH

Model	AUC	Sensitivity	Specificity	PPV	NPV
**XGBoost**	**0.761 (0.742- 0.765)**	**57.64% (57.16–58.12)**	**81.43% (80.62–82.22)**	**93.13% (92.93–93.48)**	**30.29% (29.97–30.61)**
CatBoost	0.748 (0.735–0.756)	59.09% (58.61–59-58)	83.25% (82.51–83.96)	93.21% (92.93–93.48)	34.33% (34.01–34.66)
Random Forest	0.741 (0.724–0.752)	58.89% (58.40.59.37)	81.84% (81.08–82.57)	92.49% (92.20–92.78)	34.38% (34.05–34.72)
Decision tree	0.710 (0.692–0.722)	56.14% (55.64–56.63)	80.11% (79.33–80.86)	91.28% (90.96–91.59)	32.99% (32.69–33.32)
logistic regression	0.699 (0.691–0.710)	51.30% (50.81–81.80)	84.72% (84.01–85.41)	92.73% (92.41–93.04)	31.41% (31.13–31.69)

*Abbreviation*: *AUC* Area under the receiver operating characteristic curve, *PPV* Positive predictive value, *NPV* Negative predictive value, *AUH* Asia University Hospital

## Discussion

In this study, either in internal or external validation, we found that machine learning can predict which patients will have low severity with short DLOS, and the prediction models yielded high specificity and PPV. Although complete information, including that for present illness, physical examinations, laboratory data, computed tomography, and magnetic resonance imaging, may have strengthened predictive performance, obtaining such information is impractical in triage situations where streaming is initiated. To our knowledge, ours is the first study to establish a machine learning model for predicting short DLOS in an ED through the use of triage information alone.

This model can be integrated into triage to screen and stream fast track candidates. Thus, we focused on reducing the false positive value because incorrectly streaming high-severity patients into the fast track may lead to the overconsumption of nursing resources or unnecessary patient transfers to other nonfast tract units for examination or treatment. In addition, the influence of mis-streaming low-severity patients into units other than the fast track is lower. Therefore, high specificity and PPV were used, and all models yielded low sensitivity and negative predictive values.

For efficient streaming, categorizing the abundant and undifferentiated TTAS level 3 group, which comprised over 60% of total ED visits in our hospital, is crucial. The percentage of the level 3 population was similar with that of the TTAS research by Chaou et al. [12] Therefore, a wide range of severity converged in this group to inadvertently make streaming and ED resource distribution more difficult, and severely ill patients were potentially masked and delayed by large numbers of over-triage patients with lower severity. Notably, in our cohort (discharged nontraumatic patients with level 3 TTAS), 75.9% of patients were discharged is less than 4 h. Insufficient discrimination of TTAS level 3 endangers patient safety and overall ED efficiency. The model in our study was designed to better classify patients with lower severity from this diverse population.

Nonetheless, the best-predicting model is not necessarily the most clinical practicable: computational efficiency must also be considered. For example, in Table 3, with the threshold increasing from 0.85 to 0.95, both the CatBoost and XGBoost models yielded higher specificity and PPV; however, per 100 ED visits, the number of patients with a short DLOS decreased to 5.9 in XGBoost and to 1.7 in CatBoost, respectively. Thus, the ratio of identification for fast tract candidates is affected by different thresholds. The triage nurse may select and adjust the threshold according to the physician and nurse workforce at that time to avoid overloading or underutilization. Of note, the model was designed to provide decisional support rather than to completely replace the triage provider. The personnel in charge can still override the model’s suggestion based on their professional judgement.

External validation is imperative that it aims to verify the validity of models in new patients from a different population. Before using prediction model among patients in real life, external validation is indispensable. In our study, we included an independent dataset from a different level of hospital during a different period. To address the ability of generalization, the performance of external validation was not inferior to that observed in internal validation. This is the first research regarding establishing machine learning models for predicting short LOS in ED with external validation.

In the future, establishing the model for prediction of final disposition (discharge or not) is necessary, and we will build a decision support system by integrating the prediction models of disposition and DLOS. Therefore, predicting the possibility of low-severity can be executed right after the triage information is completed. Based on both subjective judgement, and the result of prediction, the triage nurse can decide whether the patient is a suitable candidate for fast track. The actual effects of these support models are needed to be verified, including prediction performance, change of triage time, and impact on ED crowding.

### Limitation

This study has several limitations. First, pediatric patients and those with trauma were not included in this study, and these groups should be analyzed separately in the future. Second, during the study period, there was no actual strategy to stream low-severity patients of TTAS level 3 in our hospital. Besides, in other research, the criteria to select fast tract candidate were either patients with only triage level 4 and 5, or existing subjective judgements which were not suitable to apply retrospectively in our study [17, 18, 33–36]. Therefore, we could not compare our models with actual ED decision nowadays. Next, although longer stay means more consumption of medical resource, further work must be done to evaluate the most appropriate cutoff point of short DLOS in our hospital [19]. Finally, we could not include other potential confounding factors, such as occupancy rate and total boarding patients, which may represent ED crowding conditions [37, 38]. Several studies have reported that overcrowding EDs may delay medical care and potentially prolong DLOS [39, 40]; thus, crowding leads to some low-severity patients being underrecognized and reduces the performance of our model. Therefore, further investigation and the addition of other confounding factors are imperative.

## Conclusion

In this research, we developed a machine learning model by applying triage information alone to predict which patients have low severity with short DLOS (< 4 h). In future research, we aim to perform external validation to generalize the models to different hospitals and integrate this system into triage for decisional support in identifying fast track candidates. Using this model may further strengthen such streaming, and a future survey of the virtual impact is required. 

## Data Availability

The datasets generated and analysed during the current study are not publicly available due to the non-disclosure agreement in IRB restrictions. However, they are available on reasonable request. Please contact Dr. Yu-Hsin Chang for details.

## Abbreviations

ED: Emergency department
CTAS: Canadian triage and acuity scale
ESI: Emergency severity index
TTAS: Taiwan triage and acuity scale
LOS: Lengths of stay
DLOS: Discharge length of stay
CMUH: China Medical University Hospital
AUH: Asia University Hospital
ICU: Intensive care unit
SBP: Systolic blood pressure
DBP: Diastolic blood pressure
BMI: Body mass index
DT: Decision tree
RF: Random forest
LR: Logistic regression
MVS: Minimal variance sampling
ROC: Receiver operating characteristics
AUC: Area under the receiver operating characteristics curve
CI: Confidence interval
PPV: Positive predictive value
NPV: Negative predictive value
COPD: Chronic obstructive pulmonary disease
